# Analysis on Deflection of Projectile Penetrating into Composite Concrete Targets

**DOI:** 10.3390/ma15227871

**Published:** 2022-11-08

**Authors:** Yingxiang Wu, Xigui Tao, Yan Liu, Qingming Zhang, Yijiang Xue

**Affiliations:** 1 Institute of Defense Engineering, AMS, PLA, Beijing 100850, China; 2 Shandong Non-Metallic Materials Institute, Jinan 250031, China; 3 State Key Laboratory of Explosion Science and Technology, Beijing Institute of Technology, Beijing 100081, China

**Keywords:** impact dynamics, analytical model of deflection, high-velocity penetration, composite concrete target, diamond-shaped moving target

## Abstract

From an offensive point of view, increasing the impact velocity of the projectile is an effective way to enlarge its penetration depth. However, as the projectile penetrates the target, there often exists an angle of attack, the resultant force on the projectile is in a different direction from that of projectile velocity, which causes the deflection of the projectile, and thus the strike effect is greatly weakened. From the other perspective, the deflection of the projectile can contribute to proactive protection of key targets from damage caused by a deeper penetration which has been an important consideration for actual protective structure. Presently, investigations on the deflection mechanism of the impact projectile are relatively few, and there is especially a lack of more comprehensive theoretical and experimental studies. In this paper, the mechanism of projectile deflection when penetrating a composite concrete target is thoroughly analyzed. The composite concrete target composed of a concrete fixed target and multiple diamond-shaped moving targets, similar to the structural system for multi-layer overlay extension, showed better anti-penetration performance in practical protective structures. The analytical model of projectile deflection during penetrating the target is established through simultaneously resolving the dynamic equations for the projectile and moving target. Penetration tests of the composite concrete target plate impacted by a 76 mm projectile were conducted to examine the effectiveness of the analytical model, where impact velocity and point and the size of the moving target were considered. On this basis, the influences of impact velocity and point on the deflection of the projectile are disclosed, and the effects of parameters of moving target are discussed. These findings can provide significant references for optimization of advanced protective structures and improvement of their anti-penetration performance.

## 1. Introduction

High-velocity penetrations of various media by projectiles are investigated extensively for the demand of civil and military applications [1,2,3,4,5,6,7,8]. Concrete as a constructional material has been extensively used in various defense applications such as command bunkers, hardened shelters, nuclear power plants, dams, and runways which may be subjected to accidental impact by dropped objects or the threat from a military projectile [9,10,11,12,13,14,15]. Resisting the penetration of high-velocity projectiles is always a difficult topic of protection engineering. In the process of projectile–target interaction, the projectile is usually set to penetrate into the target as deeply as possible. On the contrary, reducing the penetration depth of the projectile and preventing piercing damage is the design goal of protective structures [6,16,17,18,19]. To achieve this goal, the general concept is constructing a special structure using concrete with high strength and performance, which can increase the resistance of the target to the projectile, and thus decrease the projectile penetration ability and improve its anti-penetration protection. However, the modification of concrete performance is more difficult than increasing the projectile velocity, and it is less meaningful to resist high-speed impact just by increasing the thickness of the protective structure. Although some composite concrete protective structures appear [20,21,22,23,24], the essence of them to realize anti-penetration protection is by upgrading the penetration resistance of the target.

In the last few decades, extensive penetration experiments have been carried out to improve our understanding of the problem of a concrete target penetrated by a rigid projectile and enhance our capacity to predict the ballistic performance of the projectile within the concrete target and further understand its penetration mechanism [25,26,27,28,29,30,31]. The related experimental research indicates that the projectile may undergo great changes in attitude and trajectory during penetration in the case of normal penetration with angle of attack, as the position of the part of the projectile subjected to non-axisymmetric force relative to the center of mass changes [32,33,34,35,36,37]. Based on this fact, the deflection mechanism of the projectile can be utilized to design the protective structures for better anti-penetration performance, considering that the alteration of the attitude and trajectory of the projectile during projectile–target interaction can result in prolonged penetration path, weakened penetration ability, and thus reduced penetration depth, so that the damage to the target plate can be prevented [38,39,40,41,42]. However, research on the theoretical study of the deflection mechanism in the process of the projectile impacting the concrete target has not been sufficiently detailed, and the composite concrete targets penetrated by the projectile have been less studied.

In this study, the mechanism of projectile deflection when penetrating a composite concrete target is thoroughly analyzed. The composite concrete target composed of a concrete fixed target and multiple diamond-shaped moving targets, similar to the structural system for multi-layer overlay extension, showed better anti-penetration performance in practical protective structures. The analytical model of projectile deflection during penetrating the target is established through simultaneously resolving the dynamic equations for the projectile and moving target. Penetration tests of the composite concrete target plate impacted by a 76 mm projectile were conducted to examine the effectiveness of the analytical model, where impact velocity and point and the size of moving target were considered. On this basis, the influences of impact velocity and point on the deflection of the projectile are disclosed, and the effects of parameters of the moving target are discussed. These findings can provide significant references for optimization of advanced protective structures and improvement of their anti-penetration performance.

## 2. Theoretical Framework of Deflection Analysis of Projectile

### 2.1. Composite Target Plate

**Figure 1:**
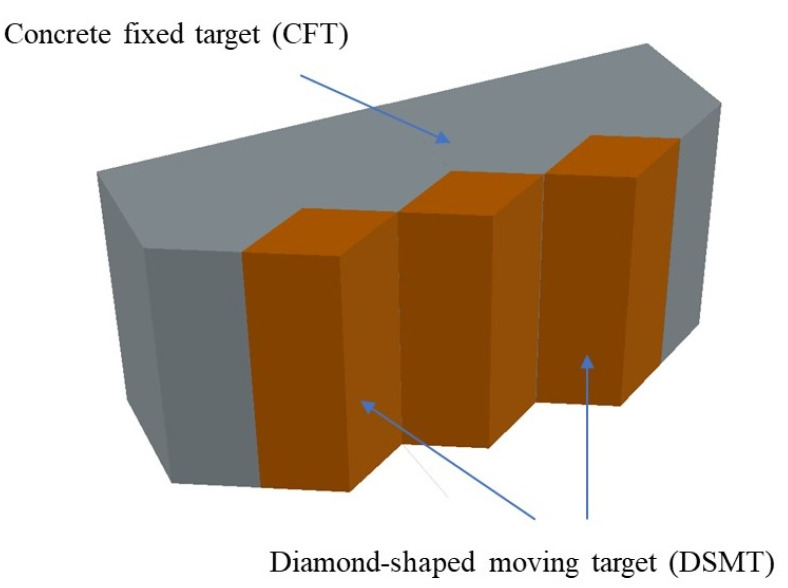
Schematic diagram of composite concrete target plate with diamond-shaped moving targets.

**Figure 2:**
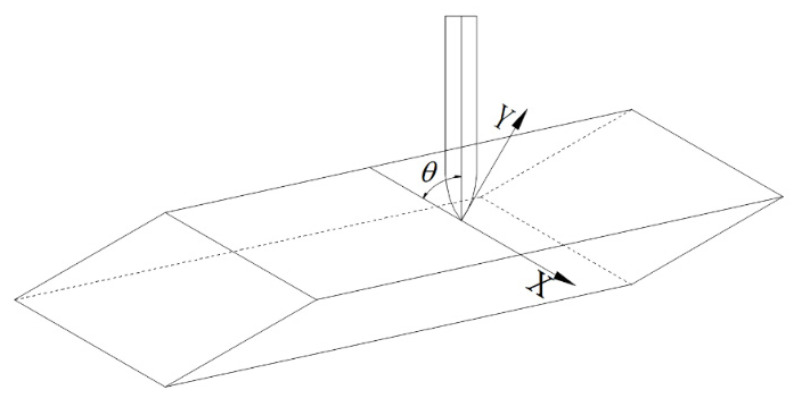
The coordinate system of the projectile penetrating the diamond-shaped moving target.

The composite concrete target consists of multiple diamond-shaped moving targets and a concrete fixed target, as shown in Figure 1. The coordinate system of the projectile penetrating the diamond-shaped moving target is presented in Figure 2. The coordinate origin represents the impact point of the projectile, and the *x*-axis is parallel with the penetrated surface of the diamond-shaped moving target. θ is the angle between the projectile and the penetrated surface.

The dynamic analysis on the projectile penetrating the diamond-shaped moving target is based on several basic assumptions. During the penetration process, the projectile is assumed to be a rigid body, and the mass losses caused by the deformation of the projectile and the erosion of the projectile are not considered. In addition, the moving target is in a free state. When the projectile penetrates the target plate, assuming that the angle of attack is zero, the influencing factors such as gravity, air resistance, and the friction induced by the projectile hitting the target are ignored. Instead, the recovery coefficient *e* is applied to take the above factors into consideration synthetically. In addition, the projectile body does not rotate, and the axis of the projectile, the speed direction, and the deflection torque are coplanar at the moment of impact.

### 2.2. Dynamic Equation of Projectile

**Figure 3:**
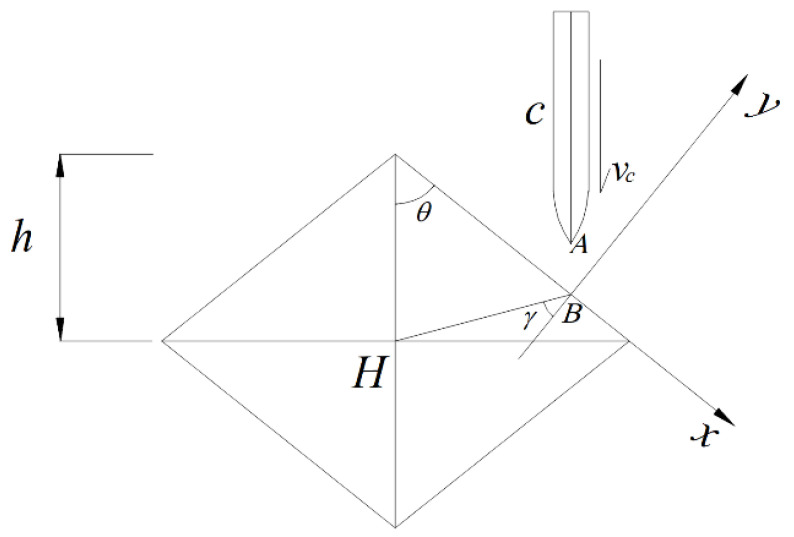
Schematic diagram of kinematic models of the projectile and the moving target before impact.

**Figure 4:**
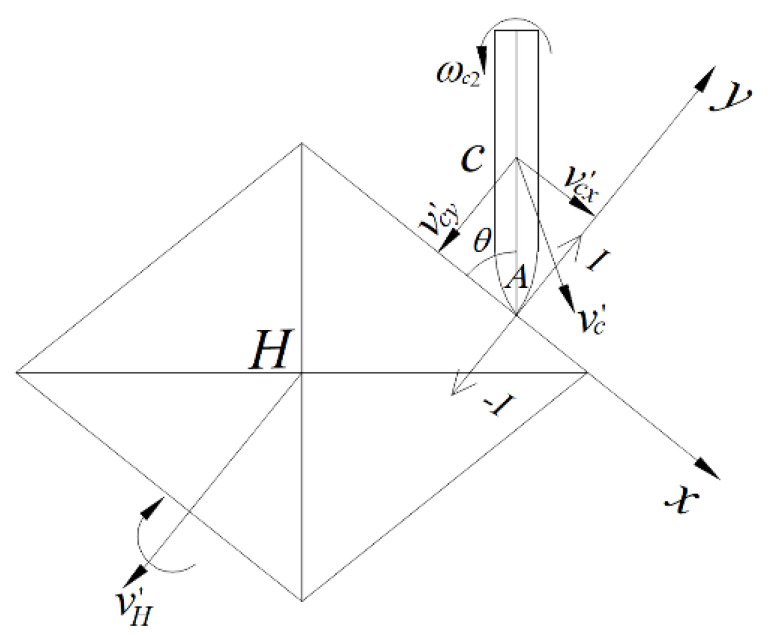
Schematic diagram of kinematic models of the projectile and moving target after impact.

The diamond-shaped moving target has central symmetry, so only one of its four surfaces is analyzed. The kinematic models of the projectile and the diamond-shaped moving target before and after the impact are respectively displayed in Figure 3 and Figure 4. The center of mass of the diamond-shaped target is *H*, and its mass and moment of inertia are, respectively, $m_{H}$ and $J_{H}$. The distance between its centroid and top is h, where the top indicates the point of the spire. The centroid of the projectile is *c*, the mass of the projectile is *m*_c_, and the moment of inertia of the projectile around its centroid is $J_{c}$. The angular velocity of the projectile, $\omega_{c1}$, before the impact is 0, and its velocity is $v_{c}$, which is equal to its flight speed.

The diamond-shaped moving target is in a free state, having no constraints. The velocity and angular velocity of the diamond-shaped target are, respectively, $v_{H}$ and $\omega_{H1}$, and both equal to 0 before impact. The distance between the head and centroid of the projectile is *AC*, equal to l. The angle between the projectile body and the penetrated surface of the diamond-shaped target before impact is *θ*. The impact point is *B*, and the angle between *HB* and the *y*-axis is $\gamma_{i}$. The velocity of the center of mass of the diamond-shaped target after the impact is ${v^{\prime }}_{H}$, and the corresponding angular velocity is $\omega_{H2}$. After impact, the velocity of the projectile is ${v^{\prime }}_{c}$ and its angular velocity is $\omega_{c2}$.

Based on the momentum and the angular momentum theorem, the dynamic equations of the projectile can be expressed as:

(1) $$m_{c}{v^{\prime }}_{cx}-m_{c}v_{cx}=\sum I_{x},$$

(2) $$-m_{c}{v^{\prime }}_{cy}-m_{c}v_{cy}=\sum I_{y},$$

(3) $$J_{C}\omega_{c2}-J_{C}\omega_{c1}=\sum M_{C}(I^{e}),$$

where $v_{cx}$ and $v_{cy}$ are, respectively, the *x*-axis and *y*-axis components of the velocity of mass center of the projectile before impact; ${v^{\prime }}_{cx}$ and ${v^{\prime }}_{cy}$ are the *x*-axis and *y*-axis components of the velocity of the center of mass of the projectile after impact, respectively; *I_x_* and *I_y_* are the *x*-axis and *y*-axis impulse of the projectile during impact, respectively; $\sum M_{c}\;\left(I\;\left(e\right)\right)$ is the sum of the moments of the impulses with respect to the center of mass of the projectile.

Ignoring the influence of friction resulting from the impact, and supposing that the projectile is affected by the impact impulse along the *y*-axis direction, *I_y_* and *I_x_* = 0, thus ${v^{\prime }}_{cx}=v_{cx}$ can be obtained. The velocity of the center of mass of the projectile along the *x*-axis direction after the impact, ${v^{\prime }}_{cx}$, is:

(4) $${v^{\prime }}_{cx}=v_{cx}=v_{c}\cos\theta .$$

The velocity of the warhead after the impact, ${v^{\prime }}_{A}$, is:

(5) $${v^{\prime }}_{A}={v^{\prime }}_{c}+{v^{\prime }}_{Ac},$$

where ${v^{\prime }}_{c}$ is the velocity of center of mass of the projectile after impact; ${v^{\prime }}_{Ac}$ is the velocity of the warhead relative to the center of mass of the projectile after impact. As the *y*-axis projection of warhead velocity of the projectile, ${v^{\prime }}_{Ay}$, causes its deflection, thus:

(6) $${v^{\prime }}_{Ay}={v^{\prime }}_{cy}+l^{\prime }\cos\theta \cdot \omega_{c2},$$

where ${v^{\prime }}_{cy}$ is the *y*-axis component of the center of mass velocity of the projectile after impact.

### 2.3. Dynamic Equation of Diamond-Shaped Moving Target

During the penetration process, the impulse of the diamond-shaped moving target is equal to the impulse of the projectile and the opposite direction, and the following can be obtained:

(7) $$m_{c}{v^{\prime }}_{cx}-m_{c}v_{cx}=\sum I_{x},$$

(8) $$-m_{c}{v^{\prime }}_{cy}-m_{c}v_{cy}=\sum I_{y},$$

(9) $$J_{C}\omega_{c2}-J_{C}\omega_{c1}=\sum M_{C}(I^{e}),$$

where $v_{Hx}$ is the component of the centroid velocity of the diamond-shaped moving target in the *x*-axis direction before impact; $v_{Hy}$ is the component of the *y*-axis velocity of the centroid velocity of the diamond-shaped moving target in the direction of the *y*-axis before impact; $v_{Hx}^{\prime }$ is the component of the diamond-active bull’s centroid velocity in the *x*-axis direction after impact; $v_{Hy}^{\prime }$ is the component of the centroid velocity of the diamond-shaped moving target in the *y*-axis direction after impact; *I_x_* is the impact impulse of the diamond-shaped moving target in the *x*-axis direction during the impact; *I_y_* is the impact impulse of the diamond-shaped moving target in the direction of the *y*-axis during the impact; $\omega_{H1}$ is the angular velocity of the diamond-shaped moving target before the impact; $\omega_{H2}$ is the angular velocity of the diamond-shaped moving target after the impact; ∑*M_H_*(*I*^(*e*)^) is the sum of the impulse moments of the impact impulse against the diamond-shaped moving target.

The diamond-shaped moving target is only affected by the impact impulse *I_y_* in the y direction, *I_x_* = 0, then it can be obtained that:

(10) $$v_{Hx}^{\prime }=v_{Hx}=0.$$

The velocity $v_{B}^{\prime }$ of the post-impact diamond-shaped moving target impact point B is equal to the vector sum of the velocity $v_{H}^{\prime }$ of the diamond-shaped moving target after the impact and the velocity $v_{BH}^{\prime }$ of the post-impact impact point B relative to the diamond-shaped moving target H is

(11) $$v_{B}^{\prime }=v_{H}^{\prime }+v_{BH}^{\prime }.$$

The distance *HB* between the diamond-shaped moving target and the impact point B is: *HB* = *hsinθ*/*cosγ*, and the *γ* is the angle between *HB* and the *y*-axis direction. If the component of the impact impulse *I* perpendicular to the *HB* direction is *Isinγ*, then the velocity $v_{B}^{\prime }$ of the diamond-shaped moving target impact point B after the impact is projected as follows:

(12) $$v_{By}^{\prime }=v_{Hy}^{\prime }+h\cdot \sin\theta \cdot \tan\gamma \cdot \omega_{H2}.$$

### 2.4. Deflection Model

The process of action of projectiles and diamond-shaped moving target involves complex deformation and damage of materials, and in order to simplify the problem, the degree of energy dissipation or change in the state of motion of the object before and after the impact can be described by the impact recovery coefficient [32,33]. Here, the impact recovery factor *e* is defined as the ratio of the separation velocity of an object after impact to the approaching velocity before impact, and expressed as:

(13) $$e=\frac{v_{By}^{\prime }-v_{Ay}^{\prime }}{v_{c}\sin\theta },$$

in which $v_{By}^{\prime }$ is the *y*-axis projection of the velocity of the diamond-shaped moving target after impact at the impact point *B*; $v_{Ay}^{\prime }$ is the *y*-axis projection of the velocity of the projectile warhead after impact.

A set of equations can be obtained by combining Equations (2), (3), (6), (8), (9), (12) and (13), and presented as:

(14) $$\{\begin{array}{l} m_{c}{v^{\prime }}_{cy}-m_{c}v\sin\theta =I\\ J_{c}\omega_{c2}=I\cdot l^{\prime }\cdot \cos\theta \\ m_{H}{v^{\prime }}_{Hy}=-I\\ J_{H}\omega_{H2}=-I\cdot h\sin\theta \cdot \tan\gamma \\ {v^{\prime }}_{By}-{v^{\prime }}_{Ay}=ev\sin\theta \end{array}.$$

The solution of the above equations is:

(15) $$\{\begin{array}{l} {v^{\prime }}_{Hy}=\frac{v\sin\theta \left(1+e\right)}{1+\frac{m_{H}{\left(h\sin\theta \cdot \tan\gamma \right)}^{2}}{J_{H}}+\frac{m_{H}}{m_{c}}+\frac{m_{H}{\left(l^{\prime }\cdot \cos\theta \right)}^{2}}{J_{c}}}\\ \omega_{c2}=-\frac{l^{\prime }\cdot \cos\theta \cdot m_{H}}{J_{c}}\cdot {v^{\prime }}_{Hy}\\ {v^{\prime }}_{cy}=-\frac{m_{H}}{m_{c}}\cdot {v^{\prime }}_{Hy}+v\sin\theta \\ \omega_{H2}=\frac{m_{H}\cdot h\sin\theta \cdot \tan\gamma }{J_{H}}\cdot {v^{\prime }}_{Hy}\\ I=-m_{H}\cdot {v^{\prime }}_{Hy} \end{array}.$$

Based on Equation (15), the respective motion parameters of the projectile and the diamond-shaped moving target can be solved after the impact between the projectile and the diamond-shaped moving target, and the respective motion trajectories can be calculated. During actual target contact, the structure will be limited in the *y* direction (the same as the direction of the flight projectile), i.e., the ${v^{\prime }}_{Hy}$ is small or equal to 0. Accordingly, the above equation can be expressed as:

(16) $$\{\begin{array}{l} {v^{\prime }}_{cy}=-\frac{v\sin\theta \left[e-\frac{m_{c}{\left(h\sin\theta \cdot \tan\gamma \right)}^{2}}{J_{H}}-\frac{m_{c}{\left(l^{\prime }\cos\theta \right)}^{2}}{J_{c}}\right]}{\frac{m_{c}{\left(h\sin\theta \cdot \tan\gamma \right)}^{2}}{J_{H}}+1+\frac{m_{c}{\left(l^{\prime }\cos\theta \right)}^{2}}{J_{c}}}\\ \omega_{c2}=\frac{m_{c}l^{\prime }\cos\theta }{J_{c}}\left({v^{\prime }}_{cy}-v\sin\theta \right)\\ \omega_{H2}=-\frac{m_{c}h\sin\theta \cdot \tan\gamma }{J_{H}}\left({v^{\prime }}_{cy}-v\sin\theta \right) \end{array}.$$

## 3. Penetration Tests

### 3.1. Target and Projectile

**Figure 5:**
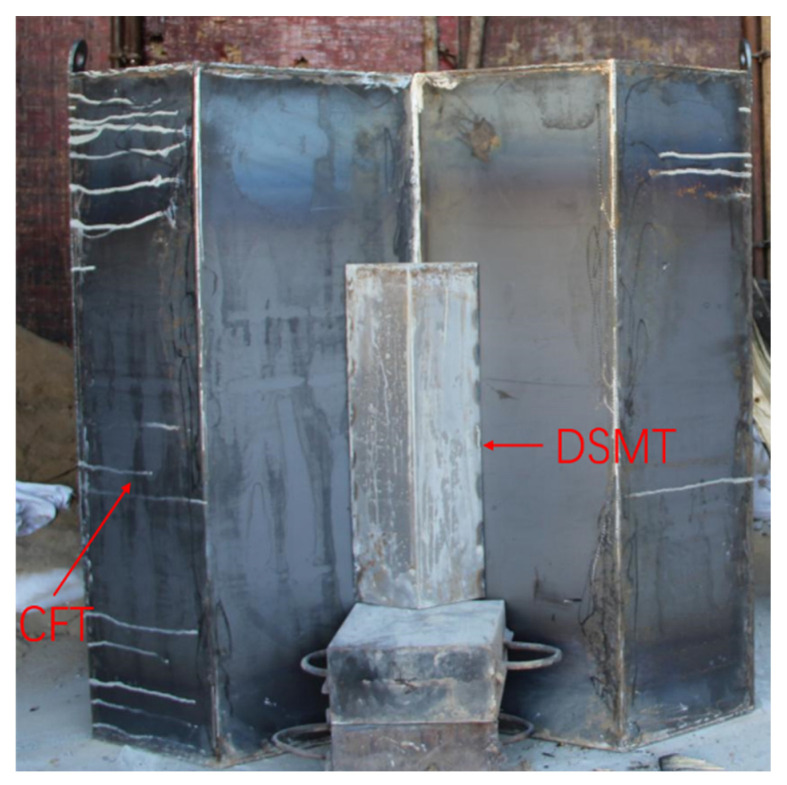
Photograph of the composite target plate with diamond-shaped moving target.

**Figure 6:**
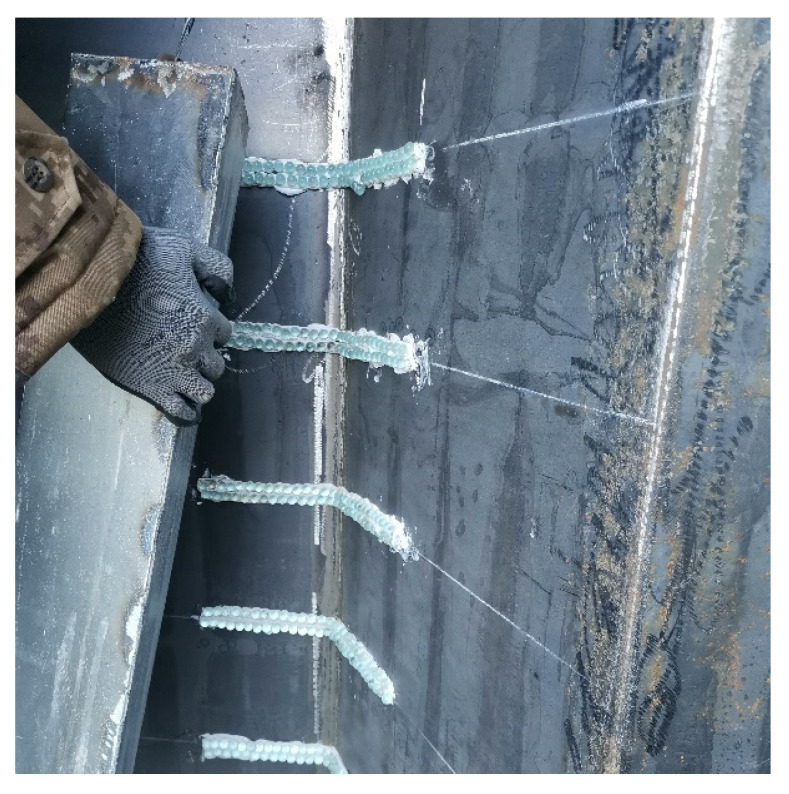
The glass ball sliding track arranged on the contact surface with the fixed target.

In order to verify the protective effect of the diamond-shaped moving target, a test target composed of a single diamond-shaped moving target and a concrete fixed target is designed, as shown in Figure 5. The moving target is equilateral and diamond-shaped with a cross-sectional edge length of 0.152 m, a cylinder height of 0.4 m, and an angle of 120 degrees between the two faces contacting with the fixed target. The diamond-shaped moving target is covered with a 2 mm thick Q235 steel plate and filled with C60 concrete inside. The concrete fixed target is wrapped in a 2 mm thick Q235 steel plate and designed with a 120-degree chute to place the diamond-shaped moving target. The fixed target face size is 1.5 m × 1.5 m, the thickness is 0.8 m, and the C60 concrete is poured and the structural reinforcement is constructed. To reduce the friction between the moving target and the fixed target, a glass ball sliding track is arranged on the contact surface with the fixed target, as shown in Figure 6.

**Figure 7:**
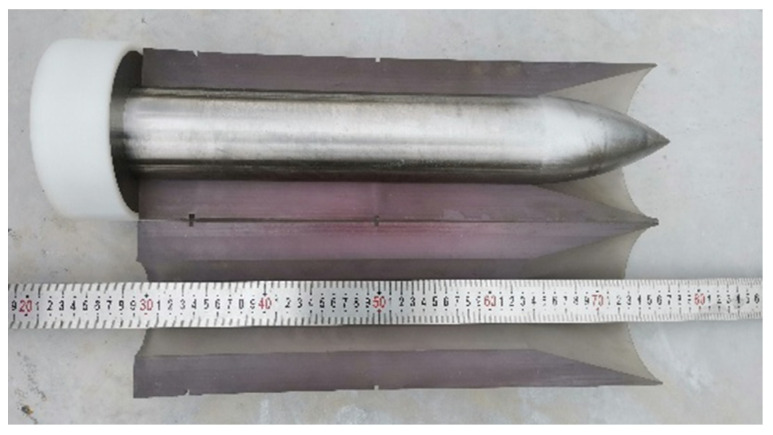
The projectile with a sabot.

The diameter of the projectile is 76 mm, and the length-to-diameter ratio is 6. The ratio of the radius of curvature of the head to its diameter is 3, and the weight of the projectile is about 14.1 kg, while the weight of the diamond-shaped moving target is about 28.2 kg, thus the mass ratio of them is 1:2. The projectile material is ultra-high-strength alloy steel 30CrMnSiNi2A, the yield strength is 1766 MPa, and the Rockwell hardness of the material after heat treatment is not less than 45. Using sub-aperture launch technology, the photo of the 76 mm test projectile body and the split-lobe stock is shown in Figure 7.

**Figure 8:**
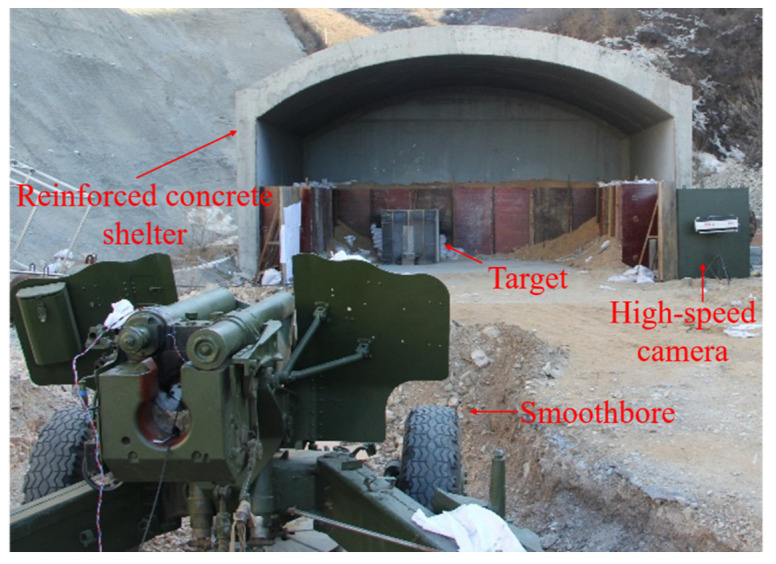
The layout of experiment site.

**Figure 9:**
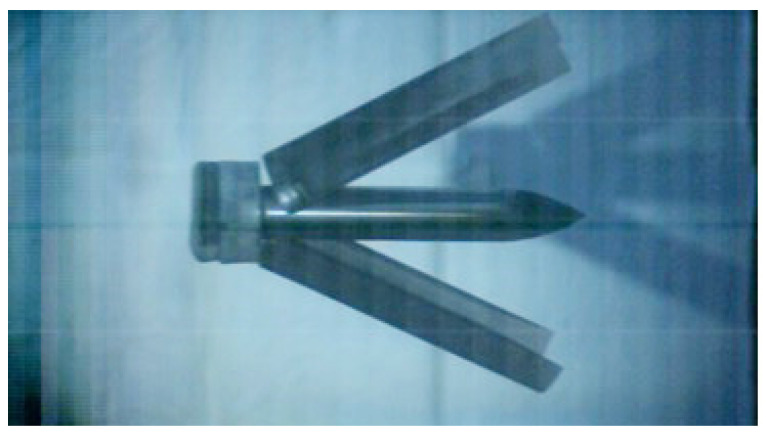
The flight attitude of the projectile.

A 140 mm caliber smoothbore gun was used as a launcher, and the test site setup is shown in Figure 8. The barrel firing direction is perpendicular to the target plate, which is placed in a concrete bunker. A high-speed camera is used to record the flight attitude of the projectile and also applied to analyze the flight speed of the projectile. The photos of the projectile body and the split-flap stock during the flight are shown in Figure 9, and the split-flap stock is separated to the sides by the action of air resistance, by which it can be seen that the projectile body has a good attitude and almost no angle of attack.

### 3.2. Experimental Arrangement

**Figure 10:**
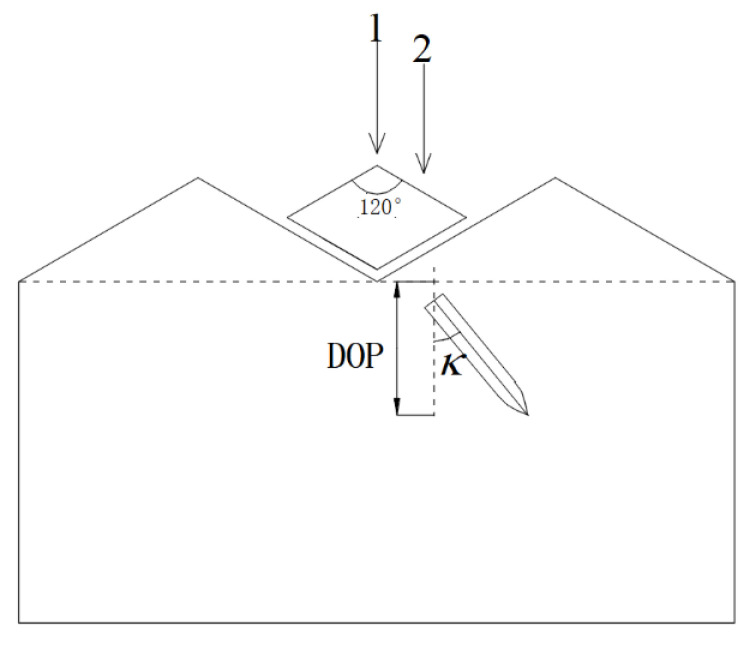
Schematic diagram of experimental measurement.

Considering that the projectile impacts different positions of the diamond-shaped moving target and the deflection effect of the projectile is different, two landing targets are designed, as shown in Figure 10, and the first landing target is located at the apex of the 120-degree angle of the diamond-shaped moving target, and the second landing target is located at the midpoint of the diamond edge. A total of 5 tests were carried out, the test conditions and test results are shown in Table 1, the definition of penetration depth and projectile deflection angle is shown in Figure 10, and the penetration depth was the vertical depth from the tip of the bullet to the target.

**Figure 11:**
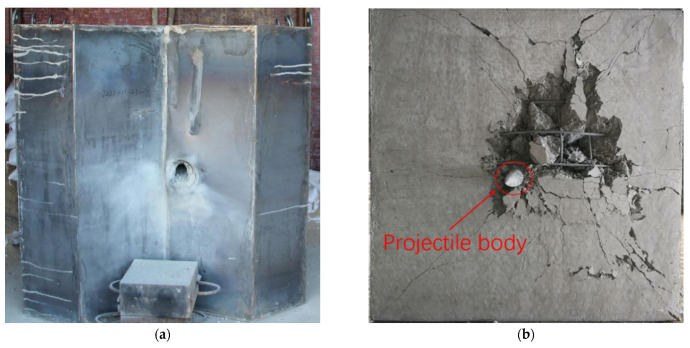
Photographs of the target plate after impact (without diamond-shaped moving target). (**a**) Front side. (**b**) Back side.

**Figure 12:**
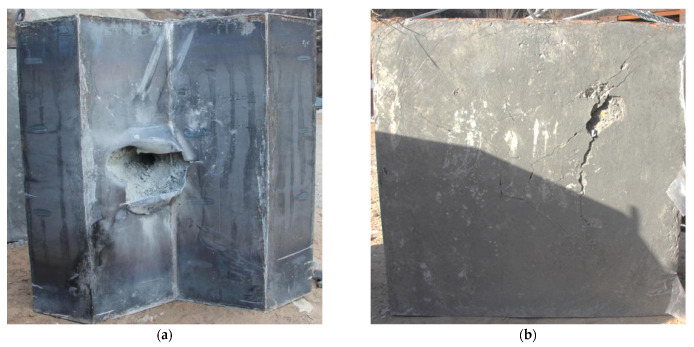
Photographs of the target plate after impact (with diamond-shaped moving target, Test 2). (**a**) Front side. (**b**) Back side.

In order to investigate the effect of the diamond-shaped moving target, the comparative experiment of Test 1 and Test 2 was carried out at impact point 2 of the target, with a velocity of about 400 m/s, and the front and back photos of the target plate after the impact are shown in Figure 11 and Figure 12, respectively. In the absence of the moving target, the tip of the projectile pierced into the target, and the projectile body deflection angle is about 3.0 degrees, as shown in Figure 11b. When the diamond-shaped moving target was arranged, the projectile did not pass through the target plate, as shown in Figure 11b, the penetration depth is 538 mm, and the deflection angle is about 22.6°. It can be detected that the diamond-shaped moving target plays an important role in deflecting the projectile.

**Figure 13:**
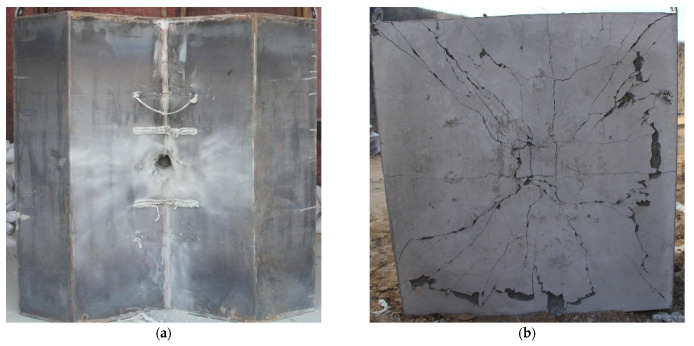
Photographs of the target plate after impact (with diamond-shaped moving target, Test 3). (**a**) Front side. (**b**) Back side.

In the case of a diamond-shaped moving target, the comparative tests of Test 3 and Test 2 were carried out at impact point 1 and 2 of the target, respectively, with a velocity of about 400 m/s, and the front and back photos of the target plate after impact are shown in Figure 12 and Figure 13, respectively. Although the projectile did not penetrate the target plate at impact point 1, as shown in Figure 13b, the projectile deflection angle was only about 15 degrees, which was significantly smaller than the result at impact point 2. From the front of the target, the projectile penetrates the diamond-shaped moving target and forms a deflection, and continues to invade the concrete fixed target at a certain angle of attack, and at impact point 2, the steel plate on the surface of the concrete fixed target is ruptured and turned outward due to the large angle of attack, as shown in Figure 12a.

In order to study the effect of the projectile velocity on the deflection of the projectile, the penetration experiments of the projectile with a velocity of 300 m/s and 500 m/s were also carried out at impact point 2, and the experiment results are shown in Table 1, and it can be seen from the final deflection angle of the projectile that the higher the elastic velocity, the more obvious the deflection effect of the diamond-shaped moving target on the projectile, indicating that the new protective structure of the diamond-shaped combined concrete proposed in this paper has achieved a better protective effect under the conditions of using ordinary materials.

## 4. Results and Discussion

### 4.1. The Influence of Impact Velocity of Projectile

**Figure 14:**
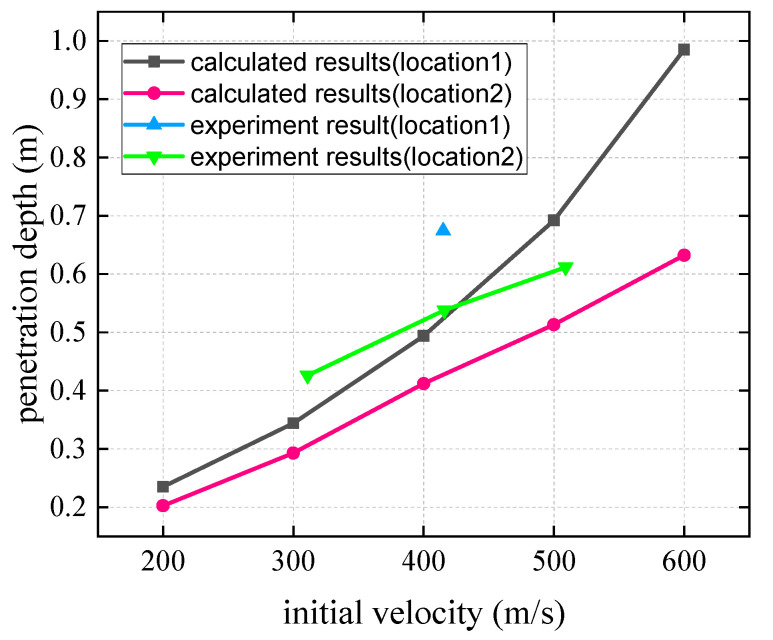
The relationship between the penetration depth and impact velocity of projectile.

**Figure 15:**
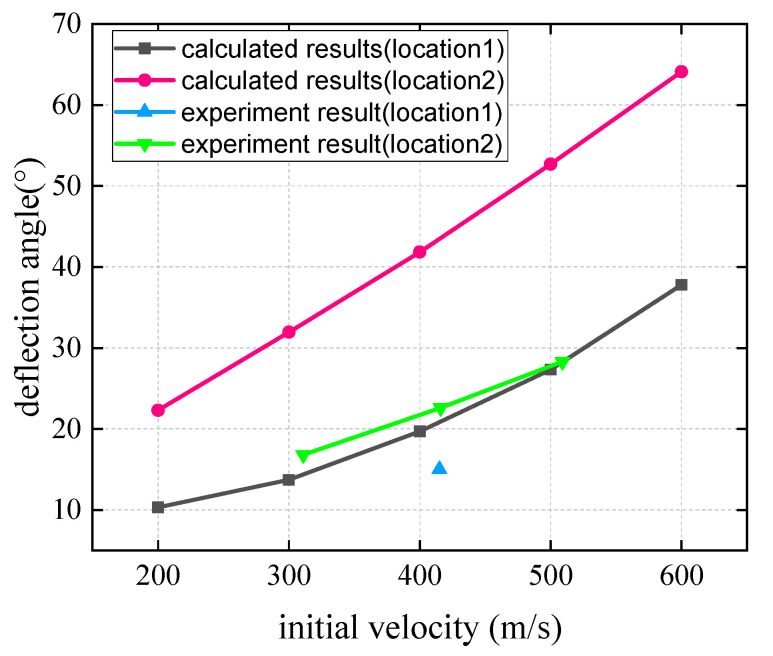
The relationship between the deflection angle and impact velocity of projectile.

For the experimental conditions in this paper, the parameters of the biased model are taken as follows: *θ* = 60 °, $J_{c}=0.2653\;\mathrm{kg}\cdot m^{2}$, $J_{H}=0.00488\;\mathrm{kg}\cdot m^{2}$, $l^{\prime }=0.228\;m$, $m_{c}=15\;k$ g, $m_{H}=5.03\;k$ g, e=−0.5, h=0.038 m, $\gamma \in \left[-\frac{\pi }{6},\frac{\pi }{3}\right]$. Combining the diamond-shaped combined target bias model obtained in this paper and the concrete fixed target plate penetration calculation method, the penetration depth and projectile deflection angles in the speed range of 200–600 m/s are calculated, and the theoretical calculation results and test results are compared with those shown in Figure 14 and Figure 15.

Theoretical calculation results show that for the two different impact positions, the depth of penetration generally increases with the increase in the initial impact velocity. When the impact velocity is identical, the depth of penetration at impact position 1 is deeper than that at impact position 2. The experimental results are largely consistent with the calculated results.

Considering the two different impact positions, the deflection angle of the projectile increases with the impact velocity. Under the same impact velocity, the deflection angle of the projectile at impact point 2 is larger than that at impact point 1. Similarly, the experimental results are in good agreement with theoretical values.

### 4.2. The Influence of Impact Position

The point B on the diamond-shaped moving target when impacted by the projectile can be any point on the *x*-axis, and the variation of the positions on the target impacted by the projectile may cause changes in the motion parameters of the projectile and diamond-shaped moving target after impact. The parameter γ is introduced to study the effect of impact point on the deflection, which is the angle between HB (the connection between the centroid of the heterogeneous structure and the impact point B) and the *y*-axis direction, as shown in Figure 3, and the value range of the γ should be —($\frac{\pi }{2}-\theta$) to θ.

**Figure 16:**
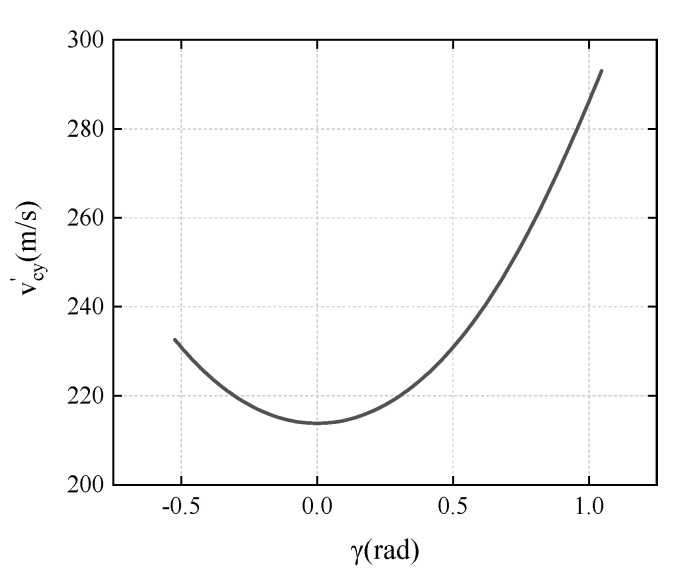
The variation of $v_{cy}^{\prime }$ with γ.

In the experimental conditions, the relationship between available $v_{cy}^{\prime }$ and γ is that:

(17) $$v_{cy}^{\prime }=\frac{427.747+297.64\tan^{2}\gamma }{0.8592\tan^{2}\gamma +1.7348}$$

and, when $\gamma \in \left[-\frac{\pi }{6},\frac{\pi }{3}\right]$, $v_{cy}^{\prime }$ shows a trend of decreasing first and then increasing as γ increases, as shown in Figure 16. The deflection angular velocity of the projectile is related to $v_{cy}^{\prime }$,

(18) $$\left|\omega_{c2}\right|=\left|6.4455\left(v_{cy}^{\prime }-346.41\right)\right|.$$

It can be seen from the above equation that when $v_{cy}^{\prime }$ < 346.41 m/s, the smaller the $v_{cy}^{\prime }$, the greater the deflection angle velocity of the projectile body, and the better the deflection effect.

From impact point 1 in Figure 10 to the bottom edge of the 60° angle of the diamond, the deflection angle of the diamond-shaped moving target on the projectile shows a tendency to increase first and then decrease, and the worst deflection effect is at the bottom edge of the 60° angle of the diamond. It can be speculated that if a hexagonal moving target is used, the target area with poor deflection effect can be significantly reduced.

### 4.3. The Influence of Moving Target

When v=400 m/s, $\gamma =-\frac{\pi }{6}$, the relationship between the $v_{cy}^{\prime }$ and the $J_{H}$ is:

(19) $$v_{cy}^{\prime }=\frac{370.936J_{H}+7.5032}{0.02166+1.7348J_{H}}.$$

**Figure 17:**
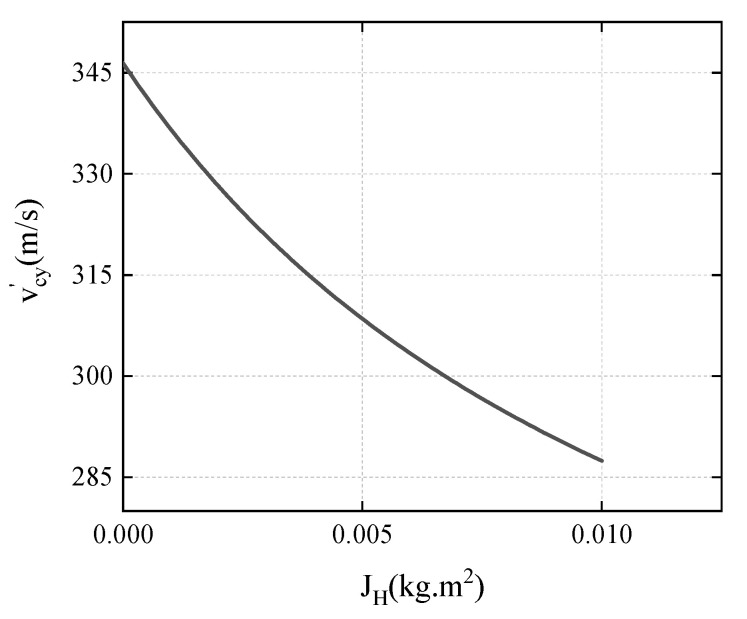
The variation of $v_{cy}^{\prime }$ with $J_{H}$.

The calculation result is shown in Figure 17, and it can be seen that as the $J_{H}$ increases, the $v_{cy}^{\prime }$ shows a decreasing trend, and the absolute value of $\omega_{c2}$ shows an increasing trend. $J_{H}$ is related to the mass and size of the diamond-shaped moving target. As the mass (size) of the diamond-shaped moving target increases, the deflection effect of the projectile becomes more obvious.

## 5. Conclusions

This study thoroughly investigated the mechanism of projectile deflection when penetrating a composite concrete target, where the composite concrete target is composed of a concrete fixed target and multiple diamond-shaped moving targets, similar to the structural system for multi-layer overlay extension, for further exploring its anti-penetration performance in practical protective structures. The analytical model of projectile deflection in the process of penetration was established through simultaneously resolving the dynamic equations for the projectile and moving target. Penetration tests of the composite concrete target plate impacted by a 76 mm projectile were conducted and compared with analytical calculations. It showed that the experimental results were largely in good agreement with calculated results, and the calculated deflection of the projectile was a little larger than in the experiment, mainly affected by the material heterogeneity.

From the comparative results of penetration in the concrete target with and without the diamond-shaped moving target, in the condition of impact velocity of about 400 m/s, the projectile was effectively deflected by the diamond-shaped moving target, where the depth of penetration into the concrete target was reduced, thus the protective performance of the target plate was promoted.

The results from experimental measurement and theoretical analysis under different impact conditions were compared, and the influences of impact velocity and point and the size of moving target on the deflection mechanism were considered. When the projectile impacted the diamond-shaped moving target with a higher velocity, the deflection effect acting on the projectile was more significant. The depth of penetration and the deflection angle generally increased with the increase in the initial impact velocity. When the impact velocity was identical, the depth of penetration at position 1 was deeper than that at position 2, and the deflection angle of the projectile at impact point 2 was larger than that at impact point 1. As the mass or size of the diamond-shaped moving target increased, the deflection effect of the projectile became more obvious.

In contrast with conventional concrete structures, the composite concrete target with a diamond-shaped moving target proposed in this paper was more economically effective in the anti-penetration of a high-speed projectile.

## Figures and Tables

**Table 1:** Experimental conditions and results.

Number	Types of Target Plates (With or Without Diamond-Shaped Moving Target)	Impact Position	Velocity of the Projectile (m/s)	DOP (mm)	Deflection Angle (Degree)
1	without	2	393	>800 (pierce)	3.0
2	with	2	416	538	22.6
3	with	1	415	674	15.0
4	with	2	311	426	16.8
5	with	2	509	612	28.3
